# Differential lateralization to faces in infants at risk of autism spectrum disorder with expressive language delay

**DOI:** 10.1002/pcn5.70054

**Published:** 2025-02-11

**Authors:** Junko Araya, Takashi Ikeda, Chiaki Hasegawa, Sumie Iwasaki, Ken Yaoi, Yuko Yoshimura

**Affiliations:** ^1^ United Graduate School of Child Development Osaka University Suita Japan; ^2^ Research Center for Child Mental Development Kanazawa University Kanazawa Japan; ^3^ United Graduate School of Child Development Kanazawa University Kanazawa Japan; ^4^ Institute of Human and Social Sciences Kanazawa University Kanazawa Japan; ^5^ Japan Society for the Promotion of Science Tokyo Japan; ^6^ Department of Psychology, Faculty of Liberal Arts Teikyo University Tokyo Japan

**Keywords:** autism spectrum disorder, face processing, infant, language, magnetoencephalography

## Abstract

**Aim:**

Face‐to‐face communication between caregiver and infant is essential for the development of language and social skills in infancy. A previous study on brain response toward human faces showed that a lateralization right fusiform gyrus (FG) response when viewing faces was associated with better social skills. However, the relationship, between infant face processing and language development remains unclear. This study aimed to examine whether brain responses to faces vary based on the ability level of language expression.

**Methods:**

Overall, 42 Japanese infants (aged 18–34 months, Mean of age (Mage) = 24.7 months, standard deviation (SD) = 4.57, 47% female) were assessed for expressive communication skills and classified into two groups: a delayed group (20 infants) and a control group (infants with typical expressive language development, 22 infants). Brain activity was recorded using a child‐customized magnetoencephalography during presentation of a mother's face, a stranger's face, and a nonface (scrambled image). The lateralization index of the FG during face viewing was calculated using the following formula: (L − R)/(L + R).

**Results:**

The results showed a significant difference in the lateralization index between the delayed and control groups. The control group showed rightward dominance of the FG activity when viewing the mother's face and others' faces, whereas the delayed group did not exhibit this lateralization. Based on behavioral observations, 75% of the delayed group met the criteria of autism spectrum disorder (ASD) risk, and infants with a high risk of ASD who had poor expressive language showed poor right hemispheric dominance compared to the control group in their brain responses to their mothers' faces.

**Conclusion:**

This study suggests that lateralization of face processing in infancy may be a predictor of expressive language abilities.

## Graphical abstract

We investigated face‐sensitive M290 in 2‐year‐old infants. Language‐delayed infants showed altered lateralization of the fusiform gyrus. Lateralization of face processing could predict language ability in infants. Infants at high risk for ASD with poor expressive language showed poor right hemispheric dominance compared to the control group in their brain responses to their mothers' faces.

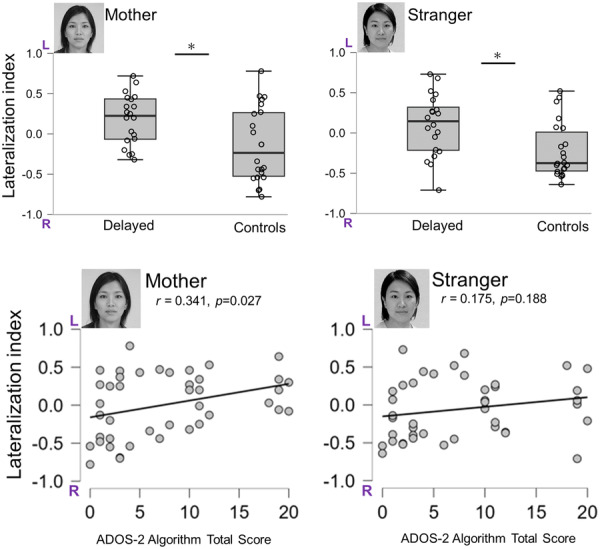

## INTRODUCTION

The typical developmental trajectory of language acquisition includes a prominent increase in the number of expressive vocabulary words between the first and second year after birth. Typical speech developmental milestones include producing words at 12 months, acquiring 50–100 words by 21 months, and forming word combinations and sentences by 24 months.^1^ However, for some children, delayed language milestones may signal persistent language impairment, affecting communication and academic progress. Infants with speech delays are at higher risk for later diagnoses of intellectual disabilities, hearing impairment, and autism spectrum disorder (ASD).^2^ Previous studies have shown a correlation between expressive language delays and joint attention deficits in infants.^3^ Joint attention, involving shared focus on an object or event with another individual through eye contact, is observed in pre‐verbal infants and is a key predictor of later language abilities,^3^, ^4^ therefore developing abilities associated with communication with others is essential for language development, and the perception of others' faces is crucial.^5^, ^6^

There seems to be a link between facial processing and language acquisition, but no empirical studies have been conducted. In neurophysiological studies, the N170/M170 component is a specific electrophysiological marker of face stimuli. This component appears in the right occipitotemporal region between 130 and 200 ms poststimulus, particularly in response to face stimuli, and has been extensively studied in adults using electroencephalography (EEG) and magnetoencephalography (MEG). In EEG and MEG studies of infants and young children, the evoked responses associated with face processing are referred to as N290/M290 and are considered precursors of the N170/M170.^7^, ^8^ In a study by Chen et al.,^6^ infants were presented with stranger face and nonface stimuli, and brain responses elicited by these stimuli were measured using MEG. A significant response (M290) was observed in the right fusiform gyrus (FG) 250–350 ms after the face presentation. This component is observed at 5 months of age and is influenced by the child's age and the type of face presented. Infants prefer their mother's face to that of a stranger until approximately 6 months of age.^9^, ^10^ Webb^11^ compared the N290 elicited by mother and stranger faces in typically developing (TD) children and children with ASD aged 18–47 months. The results showed that the amplitude of responses to stranger faces in TD children was greater than that of their mothers starting at approximately 3 years of age. However, children with ASD showed differences in their responses to mother and stranger faces at later ages compared to children in the control group. These studies suggest that the brain responses (N290) to the mother's face may be an index of the infant's developmental stage based on their autistic traits.

The human brain exhibits anatomical and functional asymmetries between the left and right hemispheres from the beginning of childhood, and avoiding the hemispheric overlap might enhance cognitive performance.^12^ This state of cerebral hemispheric dominance is called lateralization and is associated with age, language ability,^13^ and visual processing.^14^ In addition, individuals with schizophrenia and other psychiatric disorders,^15^ and those with ASD^16^ exhibit abnormalities in lateralization. The lateralization of brain responses observed in infancy is right hemispheric dominant at ∼12 months of age.^7^ Notably, several previous studies have reported an association between lateralization of face processing (right hemisphere dominance) during infancy and improved social communication skills,^7^, ^8^ but no study has examined the relationship between lateralization of face processing (i.e., right hemisphere dominance) and language development during infancy.

We aimed to investigate, using child‐customized MEG, whether the lateralization state of the response in the brain region related to face processing (i.e., FG) elicited by human faces differs based on the infant's expressive language level. We also examined whether the state of lateralization of the FG on mothers' and strangers' faces differs and whether the group with delayed expressive language includes infants at risk of ASD.

## METHODS

### Participants

Forty‐two Japanese infants (18–34 months, Mean of age (Mage) = 24.7 months, standard deviation (SD) = 4.57, 47% female) participated in this study. Inclusion criteria were (1) no clinical diagnosis of ASD; (2) Japanese as the native language; (3) no nonremovable metal in the body; (4) no hearing or visual impairments; and (5) no genetic disorders (e.g., Down's syndrome). Visual acuity was assessed using an auto‐refractometer (Spot Vision Screener; Welch Allyn Inc.). Eighteen infants did not complete the MEG measurements and 12 were excluded due to insufficient artifact‐free epochs (<30 out of 80), therefore, 20 infants (mean age = 23.3 ± 5.07 months, 7 females) were included in the expressive language delay group and 22 (mean age = 26.0 ± 3.74 months, 13 females) were included in the control group (Tables 1 and 2).

**Table 1:** Sociodemographic characteristics of participants at baseline.

Baseline characteristic	Delayed		Control	
	*ｎ*	％	*ｎ*	％
Sex				
Female	7	35	13	59
Male	13	65	9	41
ASD risks (ADOS‐2 results)	15	75	6	27

*Note*: ASD risks, children classified as having an ASD risk based on their ADOS‐2 scores in accordance with the ADOS‐2 manual.

**Table 2:** Demographic data of participant.

Measure	Delayed		Control		*t*(40) (*t*‐test)	P	Cohen's *d*
	*M* (Mean)	SD	*M* (Mean)	SD			
Age in months	23.30	5.07	26.0	3.74	1.94	0.06	0.60
ADOS‐2 Module T score	11.55	6.62	4.05	3.53	4.63	<0.001	1.43
Bayley‐Ⅲ							
Receptive communication score	5.60	2.06	8.68	1.24	5.92	<0.001	2.39
Expressive communication score	5.80	1.58	8.64	.79	7.52	<0.001	1.82
Cognitive score	8.35	1.87	9.41	1.09	2.26	0.35	0.69
Motor score	14.40	3.16	15.68	1.70	1.61	0.12	0.51

*Note*: Mean values are shown (standard deviaton [SD]). ADOS‐2 Module T, diagnostic tests to assess autism. Bayley‐Ⅲ, Bayley scales of infant and toddler development, third edition, to assess infant development. Age at time of magnetoencephalography measurement; SD, standard deviation.

### Assessments

Infant development, including motor and cognitive aspects, was assessed using the Japanese version of the Bayley Scale of Infant and Toddler Development, Third Edition (Bayley‐III).^17^ This test evaluates five areas: cognitive, language (receptive and expressive), motor, social‐emotional, and adaptive behaviors in infants aged 16 days to 42 months. Expressive language ability was assessed using the Bayley‐III Expressive Communication subscale. Participants scoring ≤7 (10th percentile) were placed in the delayed group, while others without significant delays were included in the control group. There were differences between the delayed group and the control group in the Bayley Behavior Observation Inventory. The main differences were observed in three items: “cooperation,” “distractibility,” and “fear/anxiety.” Many infants in the delayed group were less cooperative with adults' requests and tended to act at a more independent pace. Autistic traits were assessed using the Japanese version of the Autism Diagnostic Observation Schedule, 2nd edition (ADOS‐2) Module T. ASD risk was determined using two cutoff scores (8 or 10) based on language level: Algorithm 1 (12–30 months, ≤4 words, cutoff 10) and Algorithm 2 (21–30 months, ≥5 words, cutoff 8).

### MEG data acquisition

MEG recordings were conducted in a magnetically shielded room (Daido Steel Co., Ltd.). We used a 151‐channel child‐sized MEG system (PQ1151R; Yokogawa Electric Corp., Kanazawa Institute of Technology/Ricoh Co., Ltd.). Superconducting quantum interference device sensors were configured as first‐order axial gradiometers with a baseline of 50 mm. The visual stimuli were presented on a screen using a liquid crystal projector (PROPixx; VPixx Technologies Inc.). The viewing distance to the screen through the half‐mirror was 670 mm and the visual angle of the screen was 22.95 × 17.31°. The participants were placed supine on a bed and observed the screen through the half‐mirror. A charge‐coupled device camera (AS ‐808SP; Mils Systems Co., Ltd.) mounted behind the half‐mirror was set to record the participant's face during the MEG measurement. At the beginning of each session, the location of the head relative to the MEG sensors was determined based on the positions of the four marker coils (bilateral preauricular points, Cz, and 50 mm anterior to Cz) attached to the participant's scalp. Before the MEG recording, a three‐dimensional digitizer (Fastrak; Polhemus Inc.) was used to digitize the four landmarks listed above and over 200 points on the surface of the head, including the nasion and inion. During the MEG recording, two experimenters, sometimes with the participant's mother, were beside the participant to encourage them and make requests outside the shielded room regarding successful recording.

### Experimental paradigm

**Figure 1:**
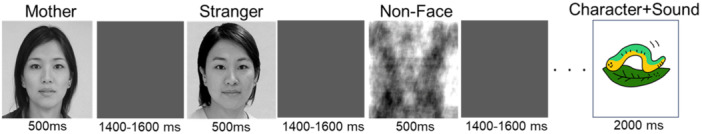
Experimental procedure: stimulus interval varied randomly between 1400 and 1600 ms. Face and nonface stimuli were pseudo‐randomized, and no stimulus was repeated. The mother stimulus was repeated 80 times, whereas the stranger and nonface stimuli were presented twice (40 per set). The order of presentation was pseudo‐randomized to prevent the same condition from occurring more than three times. For every 10 trials, a color illustration was presented with a series of five beep sounds irrelevant to the experimental task to maintain the participant's attention and motivation.

The grayscale images of faces (mothers and strangers) and phase‐scrambled images of strangers served as nonface stimuli (Figure 1). A set of neutral‐expression face stimuli of 40 nonexistent people (strangers) used in the strangers condition was generated from Generated Photos (https://generated.photos/) to match the mothers in terms of age, sex, and race. The mother's face was a grayscale photo with a neutral facial expression taken before the MEG recording. The image was cropped to the mother's face, which was equal to the stranger's face stimuli and standardized by the mean luminance distribution. Nonface stimuli were created to match the spatial frequency and luminance distribution of the strangers stimuli. This Fourier‐transform surrogate method was used by Halit, Csibra,^18^ and Chen et al.^7^ However, we did not crop images at the face contour because there was slight inhomogeneity in the luminance distribution of the facial background. The mean luminance of the stimuli and background was 201.24 and 4.12 cd/m^2^, respectively, measured using a colorimeter (CS‐150; Konica Minolta Inc.). Each image stimulus had a height and width of 600 pixels (visual angle 17.31°). The stimulus presentation was controlled using Presentation 23.1 (Neurobehavioral Systems Inc.). The duration of each stimulus presentation was 500 ms, following a blank screen, which also played a role in the baseline period of the neural response. The mean stimulus onset asynchrony was 2000 ms, with a randomjitter of 100 ms (1900–2100 ms). A trigger indicating visual stimulus onset was delivered from the PROPixx system in pixel mode with zero latency, and 80 trials were conducted for each condition (240 trials in total). The same photograph was used for all trials of the mother's face stimulus, while 40 different photographs were used for the stranger's face stimulus, each presented twice. After every 10 trials, a color illustration was presented with a series of five beep sounds irrelevant to the experimental task to maintain the participants' attention and motivation. Auditory stimuli were presented using tube‐type earphones (ER‐30; Etymotic Research Inc.) mounted on a MEG Dewar. When the participant stopped looking at the center of the screen, the experimenter paused the stimulus presentation as soon as possible, switched to attractive movies, and waited until the participant could resume the task.

### Preprocessing

**Figure 2:**
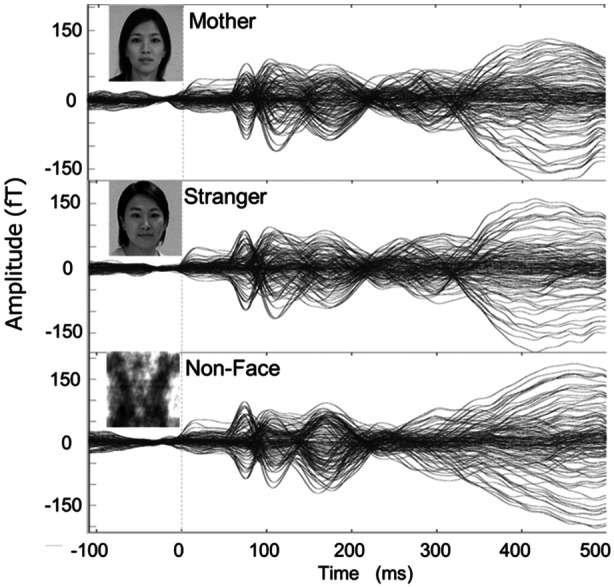
Averaged event‐related fields for all participants: event‐related fields for the mother's face, stranger's face, and nonfaces for all participants.

MEG data were measured at a sampling rate of 2000 Hz with an anti‐aliasing low‐pass filter at 500 Hz. Three sensors experienced technical problems throughout the experimental period and were excluded, resulting in 148 available sensors. Offline preprocessing and analysis of MEG data were performed using Brain toolbox^19^ in MATLAB R2022a (MathWorks Inc.). Segments containing artifacts, such as muscle activity that were difficult to remove through independent component analysis (ICA) or frequency filters, were visually inspected and excluded from further analyses. Furthermore, we extracted 20 components using ICA with the Pickard algorithm to detect eye movements and heartbeats, and removed two corresponding components from the MEG data. A band‐pass filter at 1–40 Hz was used to exclude power‐line and low‐frequency noise. The MEG data were divided into epochs from −200 to 800 ms from the onset of each visual stimulus. Epochs with excessive amplitudes, the threshold of ±4000 fT, and those when participants were not looking at the center of the screen were removed. The interval used for baseline correction was set from −50 to −0.5 ms for stimulus onset. The mean amplitude of the baseline interval was subtracted from each sensor data. Figure 2 shows event‐related fields in the mothers, strangers, and nonface conditions.

### Source estimation

**Figure 3:**
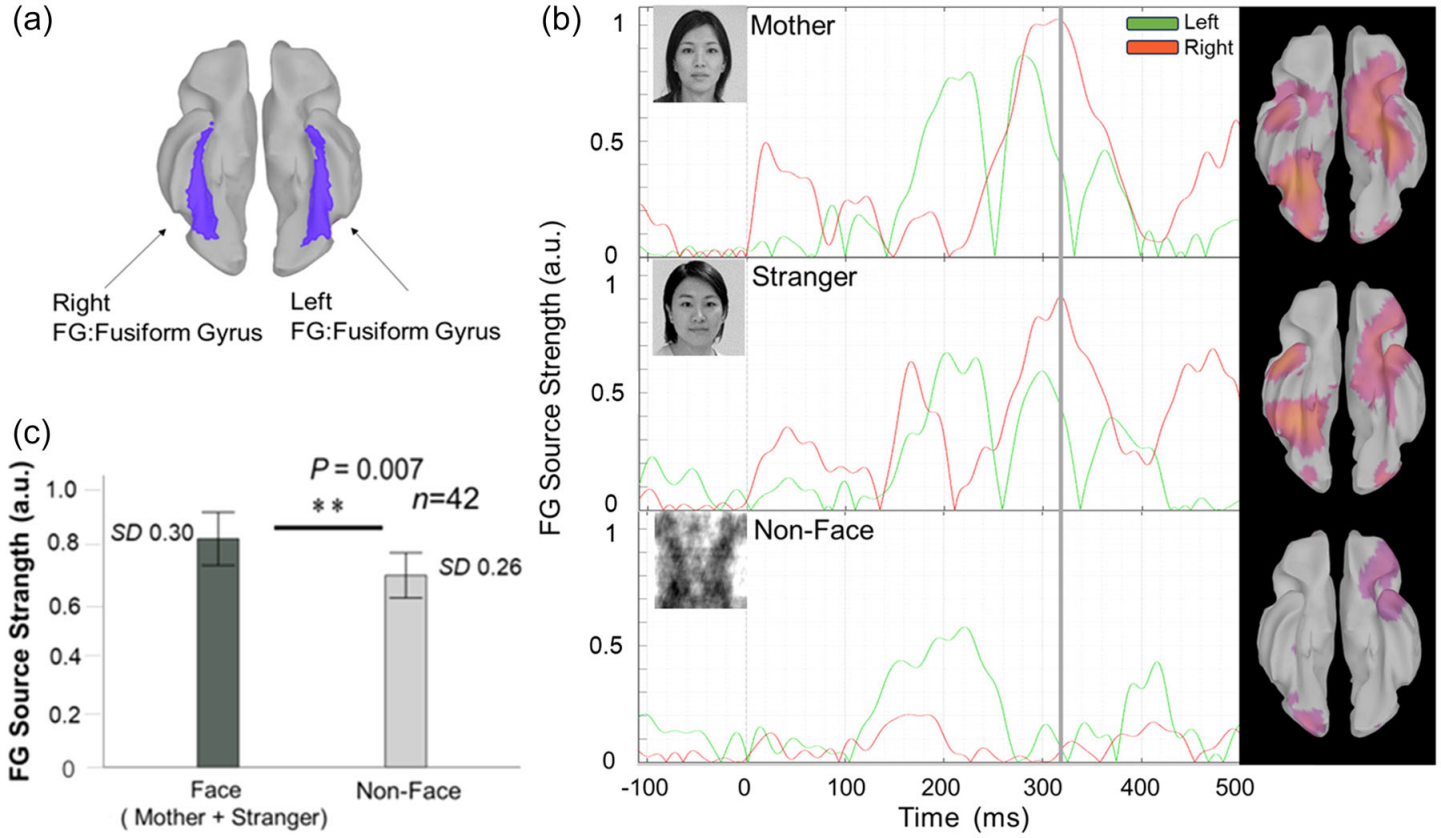
(a) This study's region of interest was the bilateral fusiform gyrus (FG) defined by the Desilkan–Killiany Atlas. (b) Averaged time courses from FG: average time courses for the right and left FG of dynamical statistical parametric mapping for the mother's face, stranger's face, and nonface conditions in a 30‐month‐old infant.(c) Face‐sensitive component (M290). According to previous studies, M290 was calculated from the 250‐350 ms interval, therefore, to validate M290, the mean of the amplitude values of the left and right FG hemispheres when looking at the face of the mother and stranger were calculated as the amplitude value. Furthermore, we examined the differences between the amplitude values for faces and nonfaces using a *t*‐test (*t* [41] = 2.97, P = 0.005), *P < 0.05.

Using the Brain toolbox, an anatomical dataset with T1‐weighted magnetic resonance imaging (MRI) structural images and cortical surface data was obtained from the brain structure template of a 24‐month‐old child.^20^ Based on the template image, each anatomical data was scaled to fit the headpoints acquired using a digitizer. MEG data were co‐registered with the anatomical data based on the four landmarks estimated using the marker coils (MEG coordinates) and determined with a digitizer (MRI coordinates), as stated in the MEG data acquisition section. The cortical surface was resampled to 15,000 vertices as current sources that were unconstrained to the orientation of the cortical mantle. An overlapping sphere–head model was created to calculate the forward solution. The noise covariance matrix was estimated by calculating sensor data from the −100 to −0.5 ms stimulus segments of the available epochs across all conditions. Furthermore, we estimated the source signal strength using the dynamical statistical parametric mapping (dSPM)^21^ method, and the source signal at each vertex was flattened by taking the norm of the three‐dimensional unconstrained vectors. We used absolute values to determine the strength of the signal, regardless of its sign. We also confirmed that the auditory‐evoked field elicited by the task‐irrelevant beep sound could be detected in the primary auditory cortex to validate the results of co‐registration and source estimation. This study's region of interest (ROI) was the bilateral FG, which was assumed to contain the fusiform face area. Vertices within the FG were specified using the Desikan–Killiany atlas^22^ (Figure 3a). All vertices in each ROI were used to calculate the mean source signal strength. The estimated amplitude of M290 as an individual index of face stimuli was taken as the maximum source strength in the time window of 250–350 ms in each hemisphere (Figure 3b). From these values, we calculated the lateralization index (LI)^7^, ^8^ according to the formula LI = (L − R)/(L + R). Positive and negative values indicate lateralization to the left and right hemispheres, respectively.

### Ethics consideration

The Ethics Committee of Kanazawa University Hospital approved the study's methods and procedures, which were performed following the Declaration of Helsinki. Parents who were fully informed of the experimental characteristics agreed to participate in the study. Written informed consent was obtained before participation.

### Statistical analysis

We used unpaired *t*‐tests and *χ* ^2^ tests to confirm no significant differences in sex, age (in months) or developmental measures between the delayed and control groups (Table 1). Furthermore, to validate the existence of the face‐sensitive M290 component, we performed a paired *t*‐test comparing the signal strength in the bilateral FG evoked by face stimuli in the mother and stranger conditions with phase‐scrambled stimuli in the nonface condition. We performed a two‐factor analysis of variance (ANOVA) to compare lateralization indices across conditions to determine whether the lateralization of the FG to faces differed between conditions in each group.

Correlation analyses based on Pearson's product–moment correlation coefficient were used to test the linear relationship between the ADOS‐2 score, severity of autistic traits, and lateralization index. The alpha level was set at 0.05 for all statistical inferences. Statistical analyses were conducted using SPSS 29.0 (IBM Corp.) and figures were drawn using JASP 0.19.0.

## RESULTS

### Difference between groups for each stimulus

There were no significant differences in the number of epochs analyzed under each condition (mother's face [delayed group: mean ± SD = 57.65 ± 15.40, control group: mean ± SD = 65.13 ± 11.66], stranger's face [delayed group: mean ± SD = 57.60 ± 15.58, control group: mean ± SD = 65.13 ± 11.24], and nonface [delayed group: mean ± SD = 58.15 ± 15.85, control group: mean ± SD = 65.86 ± 11.46] in each group (delayed group: *F* [2, 80] = 0.551, P ＝ 0.578, control group: *F* [1, 40] = 3.377, P ＝ 0.074).

### Validation of face‐sensitive M290

Previous studies have reported that the infant face processing component M290 appears at 250–350 ms after the onset of a face stimulus.^7^, ^11^ We checked whether bilateral FG ROIs were more activated during the observation of face stimuli than the nonface stimuli in this time window (Figure 3c). The source strength in each ROI estimated using dSPM under the three conditions were as follows: mother: left mean ± SD = 0.39 ± 0.22, right mean ± SD = 0.42 ± 0.25; stranger: left mean ± SD = 0.39 ± 0.21, right mean ± SD = 0.47 ± 0.30; nonface: left mean ± SD = 0.35 ± 0.17, right mean ± SD = 0.35 ± 0.21. The results of a paired *t*‐test revealed that combined activations (mother and stranger) in the bilateral FG to faces were significantly higher than nonface responses that occurred 250–350 ms after face stimulation (*t* (41) = 2.97, P = 0.005).

### Lateralization index in each condition

**Figure 4:**
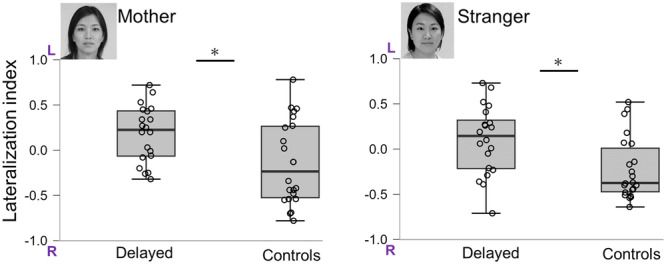
A box‐and‐whisker plot of the lateralization index using fusiform gyrus amplitude values: 20 infants in the delayed group and 22 infants in the control group. Negative values indicate right hemisphere dominance.

A two‐factor ANOVA was performed to compare lateralization indices across conditions to determine whether the FG lateralization faces differed between conditions in the delayed (mother: mean ± SD = 0.44 ± 0.193; stranger: mean ± SD = 0.47 ± 0.249) and control groups (mother: mean ± SD = 0.33 ± 0.237; stranger: mean ± SD = 0.28 ± 0.122). The results showed no significant differences between the conditions (*F* [1, 40] = 1.080, P = 305). Significant differences were found between the groups (*F* [1, 40] = 9.830, P = 0.003). The between‐group difference for the mother and stranger face conditions was P = 0.019 and 0.014, respectively. No interactions were observed (*F* [1, 40] = 0.004, P > 0.05). The control group exhibited right hemispheric dominance in FG response to face stimuli (i.e., mother and stranger). However, the delayed group showed poor right hemispheric dominance to face stimuli (mother and stranger) (Figure 4).

### Comparison of left and right hemisphere FG

Since there was a significant group difference in lateralization, we compared the activation (amplitude values) of the FG in response to face stimuli in the left and right hemispheres separately in the delayed and control groups using unpaired *t*‐test. The results showed that the delayed group had a significantly larger left hemisphere FG response to stranger faces than the control group (delayed group: left mean ± SD = 0.48 ± 0.25; control group: left mean ± SD = 0.30 ± 0.14; *t* [40] = 2.80, P = 0.009).

However, no significant differences were observed in the right hemisphere, and for responses to the mother's face, no significant differences were found in either hemisphere.

### Autistic traits and lateralization index

**Figure 5:**
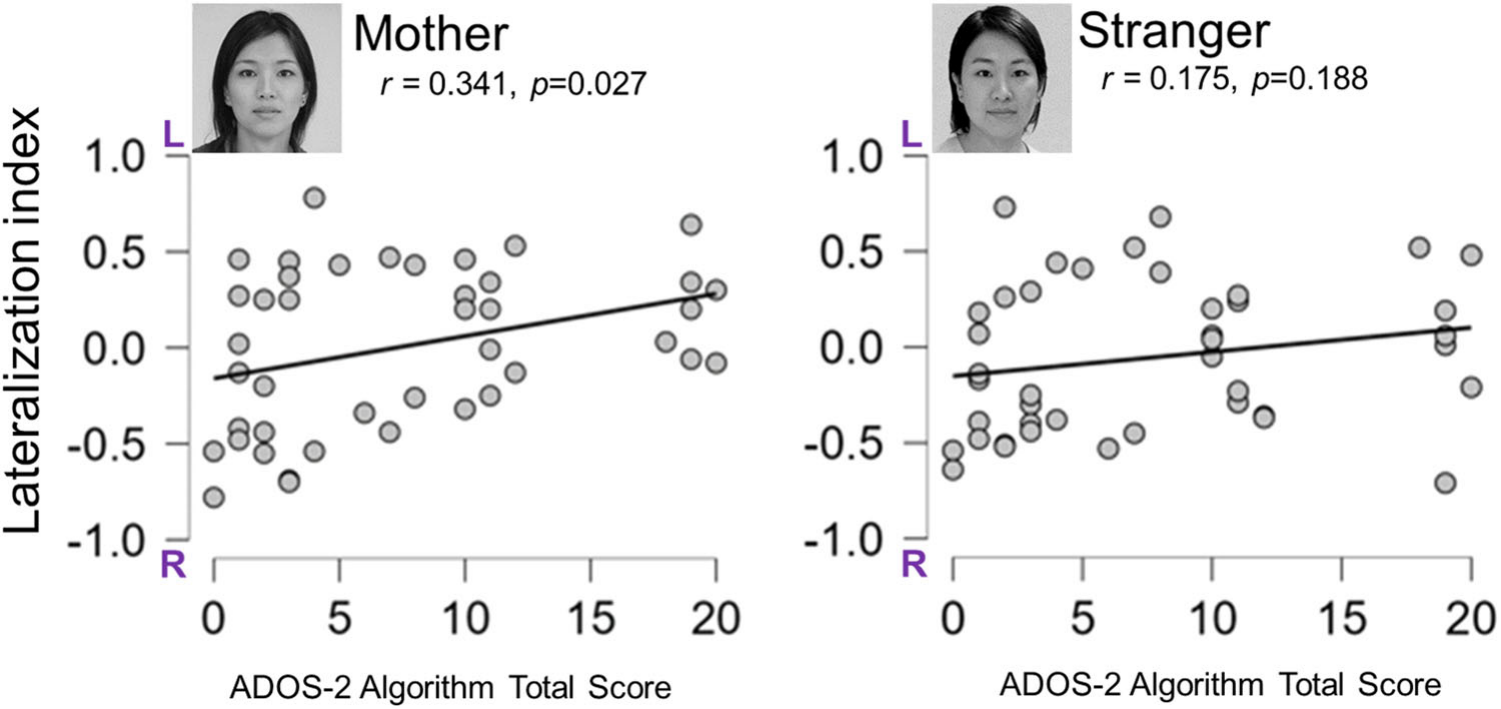
Correlation between the lateralization index and ADOS‐2 total scores for each condition for all participants (42 infants). The overall ADOS‐2 algorithm total score of the diagnostic algorithms for social effect and restricted and repetitive behaviors in Module T of ADOS‐2. Negative values indicate right hemisphere dominance. ADOS, Autism diagnostic observation schedule, 2nd edition.

The overall total score of the diagnostic algorithms Social Affect and Restricted and Repetitive Behavior in Module T of the ADOS‐2 was used. According to the Toddler Module Classification, ASD is based on two cutoff scores (8 or 10) that divide the ranges of concern. The proportion of infants at risk in each group was as follows: in the delayed and control groups, 15 of 20 infants (75%) and six of 22 infants (27%), respectively, were at risk of ASD (Table 1). We analyzed the correlation between ADOS‐2 scores (higher scores are associated with a higher risk of ASD) and the lateralization index in the mother and stranger conditions. A significant correlation was found for the mother condition (*r* = 0.341, P = 0.027), but there was no significant correlation for the stranger condition (*r* = 0.175, P = 0.188) (Figure 5).

## DISCUSSION

Joint attention involving the ability to focus on another person's face and share attention through eye contact is crucial for language development in infants.^23^ In this study, we hypothesized that face perception is linked to language development.

We aimed to investigate whether brain responses to faces differ based on the presence or absence of delays in expressive language development. Furthermore, based on previous findings involving infants, which suggest that physiological responses differ between the mother's and a stranger's face,^24^ we hypothesized that differences in brain responses to mother's versus stranger's faces are related to infants' language abilities. To investigate brain responses to faces, we used MEG to measure brain responses in the FG elicited by facial stimuli of mothers and unfamiliar individuals. Particular attention was given to the lateralization of FG responses between the left and right hemispheres. We also examined the association between the risk of ASD in infants and their face perception. The key findings of this study are as follows. First, we found that brain responses to face stimuli differed depending on the presence or absence of delays in expressive language development. In the group with delayed expressive language development, there was less lateralization of FG responses to the right hemisphere in response to face stimuli compared to the control group. Moreover, in the comparison of lateralization indices based on the type of face (mother vs. stranger), no significant differences were observed between the expressive language delay and the control groups. However, when comparing the magnitude of FG responses in each hemisphere under each condition, the expressive language delay group exhibited significantly larger FG responses in the left hemisphere to stranger faces than those of the control group. Additionally, 75% of infants in the expressive language delay group were identified as being at risk for ASD. Furthermore, analysis of the relationship between lateralization to faces and ASD risk revealed a significant correlation between the degree of right‐hemisphere lateralization of FG responses to mother faces and the level of ASD risk. This study is the first to demonstrate that the lateralization of brain activity in face perception is associated with delayed expressive language in infancy.

### The relationship between expressive language and lateralization of face processing

In this study, face‐evoked responses were analyzed in a group with delayed expressive language (*n* = 20) and a control group (*n* = 22). The control group showed right hemispheric dominance in the lateralization of FG responses to mothers' and strangers' faces. Face perception becomes right hemisphere dominant in infants aged 6–9 months.^25^, ^26^ In a study by Chen et al.,^7^ infants aged 3 months to 2 years were presented with face and nonface stimuli and a significant response was observed in the right hemisphere's FG between 250–450 ms after stimulus presentation. The present finding that infants' face‐evoked responses were right hemispheric dominant is consistent with the results of previous studies. In contrast, the delayed group exhibited poorer lateralization of brain responses to the right hemisphere when looking at a face (mother's and stranger's faces) compared with the control group. Notably, several factors may account for the lack of right hemispheric lateralization of FG responses to faces in infants with delayed expressive language. First, 75% of the delayed group in this study included infants at risk of ASD. A longitudinal study on brain responses to face stimuli in infants at risk for ASD has reported that high‐risk ASD infants, unlike TD infants, show left hemisphere dominance in face processing.^27^ Another possible factor is the atypical development of perceptual narrowing during infancy. Krasotkina and Götz^28^ stated that language and face perception may develop in parallel, with similar timing and interactions during the first year of life through the process of perceptual narrowing. Specifically, Krasotkina and Götz^28^ reported a correlation between face perception and auditory responses to language in 6‐ and 9‐month‐old infants,^28^ a process that helps infants adjust to their social group in the first 2 years of life,^6^ therefore immature perceptual narrowing in the delayed group may explain the lack of typical right‐hemispheric dominance in brain responses to face stimuli (lateralization) in infants with delayed expressive language.

### Comparison of lateralization to mother and stranger faces

This study demonstrated no significant difference in lateralization indices between mother and stranger faces in either the expressive language delay group or the control group.

An EEG study on 6‐month‐old infants reported different brain responses between their mothers' faces and strangers' faces.^29^ Similarly, another EEG study involving children aged 18–54 months found that, at approximately 24 months, brain responses to the mother's face (measured 400 ms after face presentation) were stronger than those to a stranger's face.^24^ However, after 45 months, responses to the stranger's face became stronger than those to the mother's face. Additionally, between 24 and 45 months, no significant differences were observed in brain responses to the mother's and stranger's faces, therefore the absence of a significant difference in brain responses to the mother's and stranger's faces in this study also suggests that, at these ages, infants are in a transitional phase from prioritizing recognition of the mother's face to recognizing other faces, reflecting a period of developmental change.

### FG responses to face stimuli

This study did not find a significant difference between the two groups in the lateralization of FG responses to mother or stranger faces. However, a hemisphere‐specific analysis revealed that brain responses to strangers' faces were significantly greater in the left hemisphere in the expressive language delay group. The FG includes areas that specialize in face processing and an adjacent visual word form area associated with word processing. Previous studies have shown that this region is associated with word reading after school age,^30^ semantic processing of language,^31^ second language acquisition,^32^ and other language‐related abilities. Balsamo et al.^33^ examined brain regions involved in language comprehension in 5‐ to 10‐year‐old children and reported a significant activation in the left FG associated with task accuracy. In contrast, an functional magnetic resonance imaging study on face memory in adults with prosopagnosia symptoms and age‐matched controls showed higher levels of fusiform face area activation with increasing memory load.^34^ Based on these findings and the analysis of this study, it is likely that the children with developmental delays struggled with face processing, which should have occurred effortlessly (automatically) by approximately 24 months. Children with delayed expressive language are in a state of (over) activation of the semantic processing network, including the FG, for semantic processing of mother and stranger faces; in typical development, this process should occur effortlessly (automatically) by approximately 24 months. Furthermore, among the face stimuli used in this study, the mother's face was consistent across all trials, whereas the strangers' face stimuli were different, therefore the semantic processing (labeling) and category perception of strangers' faces were more demanding than those of the “mother's faces.” This may explain why significant differences were observed, especially for stranger's faces.

### ASD risk and lateralization of the mother's face

A correlation was observed between the degree of right hemisphere lateralization to the mother's face and ASD risk (Figure 5). Additionally, many infants with ASD risk had poor expressive language, as indicated by a correlation between ADOS‐2 and Bayley expression scores. This suggest that infants at a high risk of ASD with poor expressive language exhibited poor right hemispheric dominance in their brain responses to their mothers' faces. An EEG study involving 1‐year‐old infants at high and low risk for ASD, who were presented with mothers' and strangers' faces, showed no significant difference in N290 and the late component at 400 ms after face presentation.^35^ However, a correlation was reported between the difference in brain response (400 ms after face presentation) to the mother's face versus the stranger's face in infants with a high risk of ASD and their social skills at 18 months of age,^35^ suggesting that the brain responses to the mother's face are associated with social development in infants with ASD risk.

## LIMITATIONS

This study had several limitations. First, the mother's face was repeated, while various stranger faces were shown, which could affect infants' perception due to novelty preference. Second, only faces were used, so responses to object stimuli in the FG related to semantic processing could not be assessed. Third, many infants at risk for ASD were included in the delayed language group, as ASD involves communication and speech delays. To better study expressive language delays, we suggest excluding ASD‐risk infants or focusing only on those at risk. However, excluding ASD‐risk infants may misinterpret delays as developmental, while including only at‐risk infants may link delays to ASD. Finally, although discomfort was minimized by creating a comfortable environment and excluding poor gaze or motion noise epochs, unmeasured discomfort could not be accounted for. Future studies could include physiological data (e.g., heart rate) reports on mood.

## AUTHOR CONTRIBUTIONS

Takashi Ikeda, Chiaki Hasegawa, and Ken Yaoi provided support for the MEG. All conducted the experiments. Takashi Ikeda, Chiaki Hasegawa, and Sumie Iwasaki assisted with writing, and Yuko Yoshimura provided overall support.

## CONFLICT OF INTEREST STATEMENT

The authors declare no conflict of interest.

## ETHICS APPROVAL STATEMENT

The methods were approved by the Kanazawa University Hospital Ethics Committee. They were recruited form Ishikawa and Toyama prefectures, with the research framed as a language development study.

## PATIENT CONSENT STATEMENT

Parents gave informed written consent for their children's participation.

## CLINICAL TRIAL REGISTRATION

N/A.

## Data Availability

We are open to conditional sharing; please contact the first author.
